# Informed Therapeutic Microbiome Modulation for Post-Infectious Irritable Bowel Syndrome: Pilot Experience of a Microbiome Clinic

**DOI:** 10.3390/nu18030490

**Published:** 2026-02-02

**Authors:** William Fusco, Flavio De Maio, Serena Porcari, Andrea Severino, Daniele Salvi, Stefania Piccirelli, Clarissa Ferrari, Antonio Sibilla, Gianluca Quaranta, Luca Masucci, Giovanni Cammarota, Maurizio Sanguinetti, Antonio Gasbarrini, Gianluca Ianiro

**Affiliations:** 1 Department of Translational Medicine and Surgery, Università Cattolica del Sacro Cuore, 00168 Rome, Italy; 2 Department of Laboratory and Infectious Sciences, Fondazione Policlinico Universitario Agostino Gemelli IRCCS, 00168 Rome, Italy; 3 Department of Medical and Surgical Sciences, UOC CEMAD Centro Malattie dell’Apparato Digerente, Medicina Interna e Gastroenterologia, Fondazione Policlinico Universitario Agostino Gemelli IRCCS, 00168 Rome, Italy; 4 Department of Gastroenterology and Endoscopy, Fondazione Poliambulanza Istituto Ospedaliero, 25124 Brescia, Italy; 5 Department of Medical and Surgical Sciences, UOC Gastroenterologia, Fondazione Policlinico Universitario Agostino Gemelli IRCCS, 00168 Rome, Italy

**Keywords:** microbiome, personalized medicine, precision medicine, irritable bowel syndrome

## Abstract

*Background*: Untargeted microbiome modulation has achieved conflicting results in post-infectious irritable bowel syndrome (PI-IBS). *Methods*: In a case–control study of PI-IBS patients, cases received targeted microbial modulation informed by microbiome profiling, while controls were managed by standard therapy. Treatment response was defined as a decrease in IBS-symptom severity scale (IBS-SSS) ≥50 points. *Results*: All thirteen cases (100%) and 11/20 controls (55%) experienced treatment response (*p* < 0.0001). The mean IBS-SSS score after treatments was 163 in cases and 231 in controls (*p* = 0.01). *Conclusions*: Based on our preliminary results, therapeutic microbiome modulation might be a promising strategy for PI-IBS. Further studies are needed to clarify its role.

## 1. Introduction

Irritable bowel syndrome (IBS) is one of the most prevalent chronic disorders worldwide, with a considerable social and economic burden. Gut microbiome impairment is one of the potential pathogenic pathways of IBS, especially in its post-infectious form (PI-IBS) [1].

To date, untargeted therapeutic microbiome modulation (e.g., with probiotics, antibiotics, or fecal microbiota transplantation (FMT)) has yielded conflicting results in patients with dysbiosis-associated chronic disorders, including IBS [2] and PI-IBS [3], while the inter-individual variability and the plasticity of the gut microbiome support its personalized manipulation [4,5]. A precision approach to microbial modulation implies profiling the patient’s microbiome to identify microbial targets requiring correction. Gut microbiome testing is increasingly widespread worldwide [6], and an international consensus initiative has recently regulated their provision [7]. Although there is initial evidence supporting its role in diagnosing diseases [8] and predicting response to specific therapies [9], its value in targeting gut microbiome modulation is still unknown [7]. At our Centre, we have recently established a “Microbiome Clinic”, where we manage patients with dysbiosis-associated disorders, including those with PI-IBS, with a targeted approach to microbiome modulation informed by gut microbiome testing. This approach avoids the prescription of probiotics containing bacterial species already excessively present in the patient, as well as allows the selective administration of probiotics containing the specific deficient species. Combined with pre- and antibiotics, a targeted, patient-specific gut microbiota modulation via microbiome profiling can be achieved.

Our aim was to describe outcomes of targeted microbiome modulation, informed by gut microbiome testing, in patients with PI-IBS referred to our Microbiome Clinic, compared with a control group including PI-IBS patients managed at our general Gastroenterology outpatient clinic.

## 2. Methods

### 2.1. Study Design

**Figure 1:**
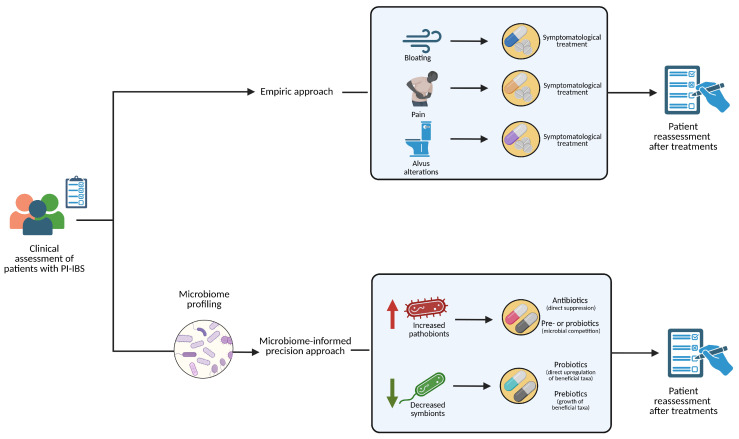
Informed therapeutic microbiome modulation (cases) vs. standard treatment (controls).

This is a pilot, prospective, single-centre, open, case–control observational study aimed at evaluating the efficacy of a precision, microbiome-based approach compared to the current empirical approach in patients with PI-IBS (Figure 1). Both cases and controls were diagnosed with PI-IBS. Cases, on one hand, received a treatment based on their fecal microbiome sequencing. At baseline, after investigating the clinical history, a fecal sample was collected for 16s-rRNA sequencing, to evaluate gut microbiota composition. Then, microbiome-modulating therapy was prescribed according to the results of the test, in order to precisely rebalance the microbiota alterations described in the test.

Controls, on the other hand, received, at baseline and after investigating their clinical history, an empirical treatment based on their symptoms and on the personal expertise of the gastroenterologist, without evaluating its gut microbiota composition. This way, a comparison between this novel approach and the current “standard of care” can be made. In both cases, general nutritionary advice was given, with the indication of a low-FODMAP diet, a temporary elimination diet based on the restriction of foods containing specific nutrients (fermentable oligosaccharides, disaccharides, monosaccharides, and polyols), known for its beneficial effect on IBS symptoms [10]. Moreover, the clinical severity of their symptoms was assessed using the IBS-SSS [11]. Then, the same score was used to evaluate the treatment response 12 weeks after the baseline visit. All these data were prospectively collected. Treatment response was defined as a decrease in IBS-SSS of at least 50 points.

### 2.2. Eligibility Criteria of Cases and Controls

Inclusion criteria for cases and controls were as follows: age ≥ 18 years old and PI-IBS diagnosis made according to the Rome-IV criteria [12]. Exclusion criteria were as follows: concomitant relevant disorders (e.g., organic chronic gastrointestinal disorders, metabolic syndrome, obesity or diabetes, gastrointestinal cancers, systemic autoimmune disorders), history of major abdominal surgery, use of antibiotics or probiotics within 4 weeks before enrollment, chronic therapy (>12 weeks) with proton pump inhibitors (PPIs), intercurrent pregnancy or lactation.

Cases were enrolled among patients referred to our Microbiome Outpatient Clinic. Controls, instead, were enrolled among patients referring to our General Gastroenterology outpatient clinic.

### 2.3. Characteristics of the Commercial Microbiome Testing

#### 2.3.1. Sample Manipulation, DNA Extraction, and Library Preparation

Danastool tubes (Danagen-Bioted, Barcelona, Spain), containing hexadecyltrimethylammonium bromide (CTAB) stabilizer, were used to collect fresh stool samples, and were stored at room temperature until processing. DNA extraction was made in a strictly controlled biosafety level 2 laboratory. In keeping with a previously described protocol, a fecal sample suspended in CTAB buffer was used to extract bacterial DNA with the DANAGENE MICROBIOME Fecal DNA kit (Danagen-Bioted, Barcelona, Spain). DNA was eluted in 200 µL of pre-heated nuclease-free water and stored at −20 °C until processing. Subsequently, DNA quantification was performed using a Nanodrop One spectrophotometer (Life Technologies, Carlsbad, CA, USA), and, once extracted, DNA was stored at −20 °C.

The V3-V4-V6 16S rRNA gene hypervariable regions were amplified using Microbiota solution B (sol.B) (Arrow diagnostics, Genova, Italy). The PCR template consisted of the extracted DNA (5.0 ng) in a 20 µL PCR volume, containing Amp Mix solutions and Enzyme Mix 1. Thermal cycling conditions were put as follows: 95 °C for 5 min; 25 cycles, each consisting of 95 °C for 30 s, 55 °C for 30 s, and 72 °C for 30 s; and, finally, 72 °C for another 5 min. Purifications of amplicons was made using the 29.5 µL Agencourt AMPure XP beads (Beckman Coulter, Brea, CA, USA), previously diluted in 19.0 µL of nuclease-free water, and were eluted in 35 µL nuclease-free water. Enzyme Mix 2 (10 µL) solution and nuclease-free water (8 µL) were mixed for each sample, and the final suspension was used to resuspend the specific lyophilized index. An amount of 2 μL of the previous amplicon was then added to every recovered index. Thermal cycling conditions were put as follows: 95 °C for 5 min; 8 cycles, each consisting of 95 °C for 30 s, 65 °C for 30 s, and 72 °C for 30 s; and, finally, 72 °C for 5 min. Purifications of indexed amplicons was made using Agencourt AMPure XP beads (Beckman Coulter, Brea, CA, USA), as already described, and they were then eluted in 25 µL of 10 mM Tris/1 mM EDTA at pH 8.0. Amplicons were then quality-checked using 1% agarose gel electrophoresis (Life Technologies, Carlsbad, CA, USA) and, using the already-mentioned method, DNA concentration was obtained. The final amplicon length was 900 bp.

The indexed amplicons of each sample were equimolarly pooled, and the final pool (5 pM) was subjected to 2 × 250 paired-end sequencing (Illumina, San Diego, CA, USA) onto an Illumina MiSeq instrument (Illumina, San Diego, CA, USA). The internal control PhiX v3 (Illumina, San Diego, CA, USA) was then introduced in the final library to enhance base diversity.

#### 2.3.2. Bioinformatic Analysis

Raw data were analyzed using the MicrobAT (Microbiota Analysis Tool) v.1.1.0 software suite provided by SmartSeq bioinformatics (Alessandria, Italy). Raw paired-end reads were cleaned to remove sequences with short read lengths (i.e., <200 nucleotides) and low quality (i.e., with an average Phred quality score of <25). Taxonomic assignment of sequences was performed by aligning all the sequences derived from the above selection process with the Ribosomal Database Project (RDP) reference database (v.11.5). The sequences that met the criteria of a minimum sequence length alignment with the RDP database of ≥80% and a similarity threshold of ≥97% were clustered at the species taxonomic level [13]. A biological observation matrix (BIOM) at different taxonomic levels was generated. Alpha diversity and relative abundances at the phylum and genus levels were subsequently computed.

### 2.4. Precision Approach

The taxonomic and ecological data obtained with gut microbiome profiling, using the method described above, were used as the basis for the patient-specific, microbiome-informed treatment. In certain cases, therapeutic modulation of gut microbiota composition was given with the aim of rebalancing the specific dysbiosis of each patient, meaning that each patient was treated differently according to their microbiome profile. This microbiome reshaping was performed according to microbiological and ecological principles, using only commercially available products.

Specifically, in the presence of an increased abundance of pathobionts, or overgrowth of non-symbiont taxa, we envisaged their direct suppression by nonabsorbable antibiotics (e.g., rifaximin, neomycin-bacitracin, and paromomycin, based on the patient’s previous therapies) [14] or, alternatively, the enhancement in beneficial taxa—to promote microbial competition against them—using prebiotics or probiotics. In cases of low α-diversity and/or decreased abundance of specific beneficial taxa, we prescribed prebiotics and/or probiotics to upregulate them, depending on the specific defect and on the commercial availability of products (e.g., in the case of low *Faecalibacterium prausnitzii* or *Roseburia intestinalis*-beneficial bacteria that produce short-chain fatty acids [SCFAs], we administered inulin to foster their growth) [15]. When relevant, based on their microbial profile, patients received antibiotics before prebiotics and/or probiotics. To minimize bloating in patients, prebiotic dosages were progressively increased up to the full dose.

### 2.5. Set of Microbiome Modulating Therapeutics

Microbiome-shaping therapies (prebiotics, probiotics, and antibiotics) and symptomatic drugs were prescribed, together with low-FODMAP dietary indications. While the drug and dosage of the symptomatic drugs were based on the clinic of the specific patient, microbiome modulators were prescribed according to microbiome testing results in cases and empirically in controls. Specifically, the prebiotics used were inulin (10 g s.i.d. for 60 days) or berberine (250 mg s.i.d. for 60 days). Probiotics were single-strain ones, like *E. coli* Nissle 1917, or multi-strain: between them, Bifidobacteria-containing multi-strain, Lactobacilli-containing multi-strain, or a combination of the two. The antibiotics used were all nonabsorbable ones, in order to limit systemic adverse effects: rifaximin (400 mg t.i.d. for 15 days), paromomycin (500 mg t.i.d. for 7 days), or a combination of neomycin and bacitracin (500 mg b.i.d. for 10 days). Alongside these microbiome therapeutics, symptomatic drugs for IBS were also prescribed: antispasmodics (otilonium bromide, octatropine methylbromide, trimebutine maleate), laxatives (macrogol 3350 and macrogol 4000), and simethicone. Dosages of the above-mentioned drugs were all predefined as the same for both cases and controls, in order to avoid disparities in treatment intensity.

### 2.6. Statistical Analysis

Continuous variables are reported as the mean ± standard deviation (SD) or median ± interquartile range (IQR), and categorical variables are expressed as frequency and percentage. Comparisons of variables, including age, sex, symptoms severity (expressed by IBS-SSS total score and subscores), IBS subtype, and treatments (type and duration) were performed using t-tests and Fisher’s exact test, as appropriate. An adjusted analysis, using multivariate linear regression, was also performed in order to evaluate the presence of any confounding factor between the data. A *p*-value of less than 0.05 was considered statistically significant. All statistical analyses were performed using SPSS v. 28.0 for Macintosh (SPSS Inc., Chicago, IL, USA).

## 3. Results

Overall, 33 patients (13 cases and 20 controls) were enrolled. The two groups were homogeneous in terms of IBS subtypes, baseline IBS-SSS scores, and demographics, except for age, as cases were younger than controls (Table 1). Moreover, no significant differences in comorbidities were observed between the two groups. Microbial alterations and related treatments of cases are reported in Table 2. Treatments administered to controls are reported in Table 3. Overall, the type and duration of treatments did not differ significantly between the two groups (Supplementary Table S1). Traditional dietary advice for IBS, without specific nutritional counselling, was given in both groups [16].

All patients in the group of cases (100%) and 11 of 20 patients in the control group (55%) experienced a treatment response (decrease in IBS-SSS score ≥ 50 points), with a significant difference between groups (*p* < 0.0001). The mean IBS-SSS score after treatments was 163 in cases and 231 in controls (*p* = 0.01). The mean reduction in IBS-SSS score was 167 in cases and 74 in the control group (*p* = 0.001). Compared with controls, cases experienced significantly lower IBS-SSS subscores after treatments for satisfaction with bowel habits (32 vs. 49, *p* = 0.03), bloating (26 vs. 43, *p* = 0.04), and quality of life (35 vs. 51, *p* = 0.009), but not for abdominal pain (29 vs. 41, *p* = 0.16) and abdominal pain frequency (30 vs. 47, *p* = 0.06). Adjusted analysis of data was also performed via multivariate linear regression. In both cases and controls, IBS subtype, age, and sex were not significantly associated with a better or worse outcome in terms of IBS-SSS total score and subscores. Thus, although not reaching statistical significance (*p* = 0.075 for cases and *p* = 0.09 for controls), female sex was associated with lower follow-up IBS-SSS than the male one. No serious adverse events were reported by any of the patients.

## 4. Discussion

In this pilot case–control study of patients with PI-IBS, targeted therapeutic microbial modulation, informed by microbiome profiling, was effective in improving the severity of IBS symptoms.

Recently, other similar pioneering strategies proved successful in targeting nutritional interventions in patients with IBS [17] and with cardiometabolic disorders [18,19]. Interestingly, the overall type of treatments did not differ between groups, supporting the hypothesis that a precise targeting of therapies, informed by microbiome, may be more effective than unsupervised approaches for PI-IBS.

We acknowledge that our study has several limitations. First, this is a preliminary observational experience of a personalized approach we apply in our Microbiome Clinic, with a limited sample size, and without a placebo control. As the microbiome usually generates hype in patients with IBS, the referral to a specialized Microbiome Clinic (which was more frequently sought by younger patients compared with controls) might have provided a placebo effect, potentially contributing to the 100% success rate in cases. As our hospital is a tertiary centre, known for its expertise in gut microbiota analysis and clinical practice, the possibility of a referral bias is another, unavoidable limitation of our study. Moreover, microbiome analysis was performed by amplicon sequencing. Although this sequencing method was allowed for commercial microbiome testing in our recent consensus initiative [7], we acknowledge that shotgun sequencing would retrieve more comprehensive data to better inform microbial-based therapies [13]. Additionally, we followed ecological principles to correct patient-specific microbial defects, but there is limited direct evidence to support this approach. Our aim to repair microbial alterations was also limited by the commercial availability of probiotics (of which most include allochthonous rather than autochthonous taxa, which are less efficient in providing a sustained microbial engraftment), as specific strains (e.g., SCFA-producers) are not commercially available [20]. We expect live biotherapeutic products to fill this therapeutic gap. Finally, we do not have post-treatment microbiome profiling, to understand whether clinical outcomes are associated with microbial shifts, as already proven for FMT [21].

These preliminary results encourage the design of placebo-controlled randomized trials, with a larger sample size, and including post-treatment microbial profiling to further explore the potential of this approach.

## 5. Conclusions

The precise modulation of specific microbial alterations, informed by microbiome testing, proved effective in reducing disease severity in patients with PI-IBS. To our knowledge, this pilot case–control study represents the first attempt made to personalize the use of microbial therapeutics according to each patient’s microbiome profile. However, further evidence is required to establish the effectiveness of this approach and properly drive clinical practice.

## Figures and Tables

**Table 1:** Demographics and baseline IBS-SSS scores of cases and controls.

Item	Cases (*n* = 13)	Controls (*n* = 20)	*p*
**Female, n (%)**	6 (46%)	13 (65%)	0.4
**Median Age (IQR)**	27 (26–36)	38.5 (17.5–59.5)	0.01
**IBS Subtypes**			
IBS-C	2	3	0.8
IBS-D	10	15	0.9
IBS-M	1	2	0.8
**Mean baseline IBS-SSS scores (±SD)**			
- Abdominal pain	60 (±20)	53 (±24)	0.4
- Abdominal pain frequency	63 (±25)	65 (±30)	0.8
- Satisfaction of bowel habits	73 (±12)	59 (±30)	0.1
- Bloating	53 (±32)	60 (±24)	0.5
- Interference with daily activities/quality of life	70 (±9)	66 (±14)	0.4
- Total score	320 (±43)	304 (±72)	0.5

**Table 2:** Microbial defects, specific treatments, and underpinning ecological principles in patients managed with a microbiome-informed precision approach.

Patient	Microbial Defects	Ecological Strategies and Related Treatment		
		Direct Suppression of Pathobionts or of Microbial Overgrowth	Direct Upregulation Of Beneficial Taxa	Symbiont Growth
#1	↑ Collinsella, Sutterella ↓ Bifidobacterium, Eubacterium, Roseburia, α-diversity	Rifaximin	Bifidobacteria-containing multi-strain probiotic	Inulin
#2	↑ Firmicutes ↓ Bifidobacterium, Butyrivibrio, Faecalibacterium, Roseburia	Rifaximin	Bifidobacteria-containing multi-strain probiotic	Inulin
#3	↓ Bifidobacterium, Butyrivibrio, Faecalibacterium, Roseburia		Bifidobacteria-containing multi-strain probiotic	Inulin
#4	↑ Enterobacteriaceae, Escherichia ↓ Akkermansia, Bifidobacterium, α-diversity	Paromomycin	Bifidobacteria-containing multi-strain probiotic	Berberine
#5	↑ Sutterella, Bacteroidaceae ↓ α-diversity	Rifaximin	Bifidobacteria- and Lactobacilli-containing multi-strain probiotic *E. coli Nissle* 1917-based probiotic	Inulin
#6	↑ Proteobacteria, Sutterella ↓ Bifidobacterium	Rifaximin	Bifidobacteria-containing multi-strain probiotic	
#7	↑ Firmicutes ↓ Bifidobacterium, Lactobacilli,	Rifaximin	Bifidobacteria- and Lactobacilli-containing multi-strain probiotic	
#8	↑ Enterobacteriaceae and Escherichia ↓ Akkermansia, Bifidobacterium, Roseburia	Rifaximin	Bifidobacteria-containing single-strain probiotic	Inulin
#9	↑ Escherichia ↓ Akkermansia, Lactobacilli	Neomycin + Bacitracin	Lactobacilli-containing multi-strain probiotic	
#10	↑ Enterobacteriaceae ↓ Akkermansia, Bifidobacterium	Neomycin + Bacitracin	Bifidobacteria-containing multi-strain probiotic	Berberine
#11	↑ Proteobacteria, Sutterella, ↓ Akkermansia, Butyrivibrio, Faecalibacterium, Roseburia	Rifaximin	Bifidobacteria- and Lactobacilli-containing multi-strain probiotic *E. coli Nissle* 1917-based probiotic	Inulin
#12	↑ Enterobacteriaceae, Desulfovibrio ↓ α-diversity, Akkermansia, Bifidobacteria, Faecalibacterium, Lactobacilli	Paromomycin	Bifidobacteria- and Lactobacilli-containing multi-strain probiotic *E. coli Nissle* 1917-based probiotic	Inulin
#13	↑ Collinsella, Escherichia ↓ Bifidobacterium, Butyrivibrio, Faecalibacterium, Roseburia	Rifaximin	Bifidobacteria-containing single-strain probiotic	Inulin

↑ increased; ↓ decreased; Specific dosages were as follows: rifaximin 400 mg t.i.d. for 15 days; paromomycin 500 mg t.i.d. for 7 days; neomycin + bacitracin 500 mg b.i.d. for 10 days; inulin 10 g s.i.d. for 60 days; berberine 250 mg b.i.d.; probiotics were all given for 60 days.

**Table 3:** Treatment of controls.

Patient	Type of Treatment						Treatment Duration (Days)
	Nonabsorbable Antibiotic	Prebiotic	Probiotics	Antispasmodic	Laxative	Simethicone	
#1	Rifaximin	Yes	Multi-strain	No	No	No	75
#2	Rifaximin	Yes	Multi-strain	Yes	No	No	75
#3	Rifaximin	No	Single-strain (Lactobacilli)	Yes	No	Yes	90
#4	No	No	Multi-strain	No	No	No	60
#5	Rifaximin	No	Multi-strain	Yes	Yes	Yes	90
#6	No	No	Single-strain (Bifidobacteria)	No	No	No	60
#7	Paromomycin	Yes *	Multi-strain	Yes	No	No	75
#8	No	Yes *	Single-strain (Lactobacilli)	No	No	No	60
#9	No	Yes	Multi-strain	No	No	No	60
#10	No	Yes *	Multi-strain	No	No	No	75
#11	Rifaximin	Yes	Single-strain (Lactobacilli)	No	No	No	90
#12	Rifaximin	Yes *	Single-strain (Lactobacilli)	No	No	No	90
#13	Paromomycin	Yes *	Multi-strain	No	Yes	Yes	75
#14	Paromomycin	No	Multi-strain	No	No	No	75
#15	Rifaximin	No	Multi-strain	No	No	No	30
#16	Rifaximin	No	Multi-strain	No	No	No	75
#17	Rifaximin	Yes *	Multi-strain	Yes	Yes	Yes	60
#18	Paromomycin	No	No	No	No	Yes	20
#19	No	No	Multi-strain	No	No	No	75
#20	No	Yes *	Single-strain (Bifidobacteria)	No	No	No	75

* As part of a synbiotic.

## Data Availability

The original contributions presented in this study are included in the article/Supplementary Materials. Further inquiries can be directed to the corresponding author.

## Supplementary Materials

The following supporting information can be downloaded at https://www.mdpi.com/article/10.3390/nu18030490/s1. Table S1. Comparison of type and duration of treatments in cases and controls.
