# Supplementary material for: Informed Therapeutic Microbiome Modulation for Post-Infectious Irritable Bowel Syndrome: Pilot Experience of a Microbiome Clinic
Source: Nutrients. 2026 Feb 2;18(3):490. doi: 10.3390/nu18030490 (PMC12899445; doi:10.3390/nu18030490)
Supplement: Supplementary file 1 [file nutrients-18-00490-s001.zip › nutrients-4099174-supplementary.pdf]

Supplementary Table S1. Comparison of type and duration of treatments in cases and controls.

	**Cases (n=13)**	**Controls (n=20)**	**P**
**Treatment type**			
Nonabsorbable antibiotics	12	13	0.07
Prebiotics	10	11	0.2
Probiotics	13	19	0.4
Antispasmodics	4	5	0.7
Laxatives	2	5	0.5
Simethicone	4	5	0.7
**Mean treatment duration (days) (±SD)**	72 (±4.8)	69 (±18.4)	0.6
